# Demonstration of reduced neoclassical energy transport in Wendelstein 7-X

**DOI:** 10.1038/s41586-021-03687-w

**Published:** 2021-08-11

**Authors:** C. D. Beidler, H. M. Smith, A. Alonso, T. Andreeva, J. Baldzuhn, M. N. A. Beurskens, M. Borchardt, S. A. Bozhenkov, K. J. Brunner, H. Damm, M. Drevlak, O. P. Ford, G. Fuchert, J. Geiger, P. Helander, U. Hergenhahn, M. Hirsch, U. Höfel, Ye. O. Kazakov, R. Kleiber, M. Krychowiak, S. Kwak, A. Langenberg, H. P. Laqua, U. Neuner, N. A. Pablant, E. Pasch, A. Pavone, T. S. Pedersen, K. Rahbarnia, J. Schilling, E. R. Scott, T. Stange, J. Svensson, H. Thomsen, Y. Turkin, F. Warmer, R. C. Wolf, D. Zhang, the W7-X Team, I. Abramovic, S. Äkäslompolo, J. Alcusón, P. Aleynikov, K. Aleynikova, A. Ali, A. Alonso, G. Anda, E. Ascasibar, J. P. Bähner, S. G. Baek, M. Balden, M. Banduch, T. Barbui, W. Behr, A. Benndorf, C. Biedermann, W. Biel, B. Blackwell, E. Blanco, M. Blatzheim, S. Ballinger, T. Bluhm, D. Böckenhoff, B. Böswirth, L.-G. Böttger, V. Borsuk, J. Boscary, H.-S. Bosch, R. Brakel, H. Brand, C. Brandt, T. Bräuer, H. Braune, S. Brezinsek, K.-J. Brunner, R. Burhenn, R. Bussiahn, B. Buttenschön, V. Bykov, J. Cai, I. Calvo, B. Cannas, A. Cappa, A. Carls, L. Carraro, B. Carvalho, F. Castejon, A. Charl, N. Chaudhary, D. Chauvin, F. Chernyshev, M. Cianciosa, R. Citarella, G. Claps, J. Coenen, M. Cole, M. J. Cole, F. Cordella, G. Cseh, A. Czarnecka, K. Czerski, M. Czerwinski, G. Czymek, A. da Molin, A. da Silva, A. de la Pena, S. Degenkolbe, C. P. Dhard, M. Dibon, A. Dinklage, T. Dittmar, P. Drewelow, P. Drews, F. Durodie, E. Edlund, F. Effenberg, G. Ehrke, S. Elgeti, M. Endler, D. Ennis, H. Esteban, T. Estrada, J. Fellinger, Y. Feng, E. Flom, H. Fernandes, W. H. Fietz, W. Figacz, J. Fontdecaba, T. Fornal, H. Frerichs, A. Freund, T. Funaba, A. Galkowski, G. Gantenbein, Y. Gao, J. García Regaña, D. Gates, B. Geiger, V. Giannella, A. Gogoleva, B. Goncalves, A. Goriaev, D. Gradic, M. Grahl, J. Green, H. Greuner, A. Grosman, H. Grote, M. Gruca, O. Grulke, C. Guerard, P. Hacker, X. Han, J. H. Harris, D. Hartmann, D. Hathiramani, B. Hein, B. Heinemann, P. Helander, S. Henneberg, M. Henkel, U. Hergenhahn, J. Hernandez Sanchez, C. Hidalgo, K. P. Hollfeld, A. Hölting, D. Höschen, M. Houry, J. Howard, X. Huang, Z. Huang, M. Hubeny, M. Huber, H. Hunger, K. Ida, T. Ilkei, S. Illy, B. Israeli, S. Jablonski, M. Jakubowski, J. Jelonnek, H. Jenzsch, T. Jesche, M. Jia, P. Junghanns, J. Kacmarczyk, J.-P. Kallmeyer, U. Kamionka, H. Kasahara, W. Kasparek, N. Kenmochi, C. Killer, A. Kirschner, T. Klinger, J. Knauer, M. Knaup, A. Knieps, T. Kobarg, G. Kocsis, F. Köchl, Y. Kolesnichenko, A. Könies, R. König, P. Kornejew, J.-P. Koschinsky, F. Köster, M. Krämer, R. Krampitz, A. Krämer-Flecken, N. Krawczyk, T. Kremeyer, J. Krom, I. Ksiazek, M. Kubkowska, G. Kühner, T. Kurki-Suonio, P. A. Kurz, M. Landreman, P. Lang, R. Lang, S. Langish, H. Laqua, R. Laube, S. Lazerson, C. Lechte, M. Lennartz, W. Leonhardt, C. Li, C. Li, Y. Li, Y. Liang, C. Linsmeier, S. Liu, J.-F. Lobsien, D. Loesser, J. Loizu Cisquella, J. Lore, A. Lorenz, M. Losert, A. Lücke, A. Lumsdaine, V. Lutsenko, H. Maaßberg, O. Marchuk, J. H. Matthew, S. Marsen, M. Marushchenko, S. Masuzaki, D. Maurer, M. Mayer, K. McCarthy, P. McNeely, A. Meier, D. Mellein, B. Mendelevitch, P. Mertens, D. Mikkelsen, A. Mishchenko, B. Missal, J. Mittelstaedt, T. Mizuuchi, A. Mollen, V. Moncada, T. Mönnich, T. Morisaki, D. Moseev, S. Murakami, G. Náfrádi, M. Nagel, D. Naujoks, H. Neilson, R. Neu, O. Neubauer, T. Ngo, D. Nicolai, S. K. Nielsen, H. Niemann, T. Nishizawa, R. Nocentini, C. Nührenberg, J. Nührenberg, S. Obermayer, G. Offermanns, K. Ogawa, J. Ölmanns, J. Ongena, J. W. Oosterbeek, G. Orozco, M. Otte, L. Pacios Rodriguez, N. Panadero, N. Panadero Alvarez, D. Papenfuß, S. Paqay, E. Pawelec, T. S. Pedersen, G. Pelka, V. Perseo, B. Peterson, D. Pilopp, S. Pingel, F. Pisano, B. Plaum, G. Plunk, P. Pölöskei, M. Porkolab, J. Proll, M.-E. Puiatti, A. Puig Sitjes, F. Purps, M. Rack, S. Récsei, A. Reiman, F. Reimold, D. Reiter, F. Remppel, S. Renard, R. Riedl, J. Riemann, K. Risse, V. Rohde, H. Röhlinger, M. Romé, D. Rondeshagen, P. Rong, B. Roth, L. Rudischhauser, K. Rummel, T. Rummel, A. Runov, N. Rust, L. Ryc, S. Ryosuke, R. Sakamoto, M. Salewski, A. Samartsev, E. Sánchez, F. Sano, S. Satake, J. Schacht, G. Satheeswaran, F. Schauer, T. Scherer, A. Schlaich, G. Schlisio, F. Schluck, K.-H. Schlüter, J. Schmitt, H. Schmitz, O. Schmitz, S. Schmuck, M. Schneider, W. Schneider, P. Scholz, R. Schrittwieser, M. Schröder, T. Schröder, R. Schroeder, H. Schumacher, B. Schweer, S. Sereda, B. Shanahan, M. Sibilia, P. Sinha, S. Sipliä, C. Slaby, M. Sleczka, W. Spiess, D. A. Spong, A. Spring, R. Stadler, M. Stejner, L. Stephey, U. Stridde, C. Suzuki, V. Szabó, T. Szabolics, T. Szepesi, Z. Szökefalvi-Nagy, N. Tamura, A. Tancetti, J. Terry, J. Thomas, M. Thumm, J. M. Travere, P. Traverso, J. Tretter, H. Trimino Mora, H. Tsuchiya, T. Tsujimura, S. Tulipán, B. Unterberg, I. Vakulchyk, S. Valet, L. Vanó, P. van Eeten, B. van Milligen, A. J. van Vuuren, L. Vela, J.-L. Velasco, M. Vergote, M. Vervier, N. Vianello, H. Viebke, R. Vilbrandt, A. von Stechow, A. Vorköper, S. Wadle, F. Wagner, E. Wang, N. Wang, Z. Wang, T. Wauters, L. Wegener, J. Weggen, T. Wegner, Y. Wei, G. Weir, J. Wendorf, U. Wenzel, A. Werner, A. White, B. Wiegel, F. Wilde, T. Windisch, M. Winkler, A. Winter, V. Winters, S. Wolf, R. C. Wolf, A. Wright, G. Wurden, P. Xanthopoulos, H. Yamada, I. Yamada, R. Yasuhara, M. Yokoyama, M. Zanini, M. Zarnstorff, A. Zeitler, H. Zhang, J. Zhu, M. Zilker, A. Zocco, S. Zoletnik, M. Zuin

**Affiliations:** 1 Max-Planck-Institut für Plasmaphysik, Greifswald, Germany; 2 Laboratorio Nacional de Fusion, CIEMAT, Madrid, Spain; 3 Laboratory for Plasma Physics (LPP), École royale militaire/Koninklijke Militaire School (ERM/KMS), Brussels, Belgium; 4 Princeton Plasma Physics Laboratory, Princeton, NJ USA; 5 Present Address: Fritz-Haber-Institut der Max-Planck-Gesellschaft, Berlin, Germany; 6 Wigner Research Centre for Physics, Budapest, Hungary; 7 Massachusetts Institute of Technology, Cambridge, MA USA; 8 Max Planck Institute for Plasma Physics, Garching, Germany; 9 University of Wisconsin Madison, Madison, WI USA; 10 Institute for Energy and Climate Research – Plasma Physics, Research Center Jülich, Jülich, Germany; 11 The Australian National University, Canberra, Australian Capital Territory Australia; 12 Technical University of Denmark, Kongens Lyngby, Denmark; 13 Eindhoven University of Technology, Eindhoven, Netherlands; 14 University of Cagliary, Cagliari, Italy; 15 Consorzio RFX, Padova, Italy; 16 Instituto de Plasmas e Fusao Nuclear, Lisbon, Portugal; 17 CEA Cadarache, Saint-Paul-lez-Durance, France; 18 Ioffe Physical-Technical Institute of the Russian Academy of Sciences, St Petersburg, Russian Federation; 19 Oak Ridge National Laboratory, Oak Ridge, TN USA; 20 University of Salerno, Fisciano, Italy; 21 ENEA Centro Ricerche Frascati, Frascati, Italy; 22 Institute of Plasma Physics and Laser Microfusion, Warsaw, Poland; 23 University of Szczecin, Szczecin, Poland; 24 University of Milano-Bicocca, Milan, Italy; 25 Auburn University, Auburn, AL USA; 26 Karlsruhe Institute of Technology, Eggenstein-Leopoldshafen, Germany; 27 National Institute for Fusion Science, Toki, Japan; 28 Universidad Carlos III de Madrid, Madrid, Spain; 29 Greifswald University, Greifswald, Germany; 30 Institute for Surface Process Engineering and Plasma Technology, University of Stuttgart, Stuttgart, Germany; 31 Austrian Academy of Science, Vienna, Austria; 32 Institute for Nuclear Research, Kiev, Ukraine; 33 Technical University of Berlin, Berlin, Germany; 34 University of Opole, Opole, Poland; 35 Aalto University, Espoo, Finland; 36 University of Maryland, College Park, MA USA; 37 Physikalisch Technische Bundesanstalt (PTB), Braunschweig, Germany; 38 Kyoto University, Kyoto, Japan; 39 Istituto di Fisica del Plasma “Piero Caldirola”, Milan, Italy; 40 Culham Centre for Fusion Energy, Abingdon, UK; 41 Los Alamos National Laboratory, Los Alamos, NM USA

**Keywords:** Plasma physics, Magnetically confined plasmas

## Abstract

Research on magnetic confinement of high-temperature plasmas has the ultimate goal of harnessing nuclear fusion for the production of electricity. Although the tokamak^1^ is the leading toroidal magnetic-confinement concept, it is not without shortcomings and the fusion community has therefore also pursued alternative concepts such as the stellarator. Unlike axisymmetric tokamaks, stellarators possess a three-dimensional (3D) magnetic field geometry. The availability of this additional dimension opens up an extensive configuration space for computational optimization of both the field geometry itself and the current-carrying coils that produce it. Such an optimization was undertaken in designing Wendelstein 7-X (W7-X)^2^, a large helical-axis advanced stellarator (HELIAS), which began operation in 2015 at Greifswald, Germany. A major drawback of 3D magnetic field geometry, however, is that it introduces a strong temperature dependence into the stellarator’s non-turbulent ‘neoclassical’ energy transport. Indeed, such energy losses will become prohibitive in high-temperature reactor plasmas unless a strong reduction of the geometrical factor associated with this transport can be achieved; such a reduction was therefore a principal goal of the design of W7-X. In spite of the modest heating power currently available, W7-X has already been able to achieve high-temperature plasma conditions during its 2017 and 2018 experimental campaigns, producing record values of the fusion triple product for such stellarator plasmas^3,4^. The triple product of plasma density, ion temperature and energy confinement time is used in fusion research as a figure of merit, as it must attain a certain threshold value before net-energy-producing operation of a reactor becomes possible^1,5^. Here we demonstrate that such record values provide evidence for reduced neoclassical energy transport in W7-X, as the plasma profiles that produced these results could not have been obtained in stellarators lacking a comparably high level of neoclassical optimization.

## Abstract (web summary)

Previously documented record values of the fusion triple product in the stellarator Wendelstein 7-X are shown to be evidence for reduced neoclassical energy transport in this optimized device.

## Main

In the quest for a viable fusion reactor, consideration of the plasma energy balance shows that—regardless of the confinement concept—a minimum value of the fusion triple product, *nTτ*_E_, must be attained before net-energy-producing operation becomes possible^1,5^. Here, *n* is the fuel density, *T* is its temperature and *τ*_E_ is the energy confinement time, defined by the ratio *W*/*P*, where *W* is the stored plasma energy and *P* is the heating power provided by fusion reactions. The temperature dependence of the fuel’s fusion reactivity provides an additional constraint; for deuterium–tritium fusion, this reactivity falls rapidly below a temperature of 10 keV (≈1.2 × 10^8^ K). High temperatures are thus mandatory in fusion plasmas but must be simultaneously consistent with a tolerable level of energy transport if the required *τ*_E_ is to be achieved.

Toroidal magnetic confinement of fully ionized fusion plasmas requires that field lines spiral around the minor axis of the torus poloidally as they encircle the major axis toroidally, tracing out magnetic flux surfaces in the course of numerous transits about the device. For a tokamak these toroidal and poloidal components of **B** are provided, respectively, by planar current-carrying coils situated outside the plasma and by a toroidal plasma current induced with a central solenoid. The strength of the magnetic field in a tokamak plasma scales inversely with the distance from the major axis of the torus and *B* ≡ |**B**| thus varies along field lines, being largest on the inboard side of the torus and smallest on the outboard side. For strongly magnetized plasmas, both the total energy and the magnetic moment are constants of the particle motion, so that particles having only a small portion of their velocity vector aligned with the magnetic field will become trapped in this variation of *B*, and in axisymmetric tokamaks they perform so-called banana orbits, which are a consequence of the vertical drift caused by the **B** × ∇*B* terms in the particles’ guiding centre equation of motion^6,7^. In the absence of collisions, these banana orbits experience no net radial displacement on average over the course of their periodic ‘bounce’ motion. However, as in the ‘classical’ case of plasma immersed in a homogeneous magnetic field, Coulomb collisions will cause such particle orbits to undergo a random-walk diffusive process with a repetition rate linearly proportional to the collision frequency, *ν*. As the ‘width’ of banana orbits is larger than the particle’s gyroradius, the resulting transport will exceed the classical level and the adjective ‘neoclassical’ is used to signify that the inhomogeneity of *B* has been accounted for^8,9^.

High-temperature fusion plasmas are characterized by low collisionality (ν*, the ratio of collision frequency to banana bounce frequency), which scales as ν* ∝ *nT*^−2^. For such plasmas, aside from the large geometrical factor due to tokamak banana orbits^9^, the neoclassical energy flux through the magnetic surface with a minor radius of *r* will obey $V{\prime} {Q}_{{\rm{neo}}}\propto {n}^{2}{T}^{1/2}{B}_{0}^{-2}$, where the left-hand side of this expression is the product of magnetic surface area, *V*′, and flux-surface-averaged neoclassical energy-flux density, *Q*_neo_, and where *B*_0_ is the average magnetic field strength at the major radius of the plasma axis (denoted by *R*_0_). This is noteworthy as these scalings are identical to those of the classical case, and the temperature dependence is therefore benign.

In the majority of tokamak experiments, energy confinement is found to be worse than predicted by neoclassical theory, which is thought to be predominantly due to plasma turbulence. If the turbulence is of gyro-Bohm nature, the energy flux will scale with *T*^5/2^ (ref. ^10^), and as neoclassical and turbulent losses are additive, the former are generally ignored when assessing the overall energy confinement to be expected in a tokamak reactor. The situation is very different in a high-temperature stellarator plasma, however, as will be discussed next.

## The need to and means of reducing the stellarator *Q*_neo_

Stellarators have the advantage over tokamaks of producing both the toroidal and poloidal components of their magnetic field with current-carrying coils external to the plasma, thereby possessing an inherent steady-state capability that tokamaks lack. Numerous possibilities exist for the placement of coils so as to provide the spiraling of field lines needed for the formation of flux surfaces, the most intuitive of which is the use of continuous helical windings as employed in heliotrons such as the Large Helical Device (LHD)^11^. Strong plasma shaping, however, is more readily produced with a set of modular coils^12^ appropriately twisted into non-planar forms, and it is this concept that underlies W7-X. In either case, the coil shapes combine both toroidal and poloidal excursions so that the magnetic field they produce cannot be axisymmetric and, indeed, an additional corrugation of *B* arises, which is commonly referred to as the stellarator’s ‘helical’ ripple. Particles that become trapped in these ripples are said to be localized, as they experience only a small variation of poloidal angle between successive reflection points and this leads to a non-zero time average of their vertical drift over this bounce period, unlike the case of tokamak banana orbits^13^. As a consequence, the collisionless trajectories of localized particles may be able to leave the plasma after numerous reflections, and confinement of such particles will occur only if collisions are frequent enough to limit their radial excursions. Collisions are thus beneficial, and in such a case, typical for electrons in high-temperature stellarator plasmas, the random-walk diffusive transport becomes inversely proportional to the collision frequency and electrons are therefore said to be in the ‘1/*ν* regime’. This regime is most remarkable for the very unfavourable temperature dependence of its neoclassical energy flux which scales as  $V{\prime} {Q}_{{\rm{neo}}}\propto {{\epsilon }}_{{\rm{eff}}}^{3/2}{T}^{9/2}{R}_{0}^{-1}{B}_{0}^{-2}$ (ref. ^14^). The quantity *ϵ*_eff_ is referred to as the effective helical ripple for 1/*ν* transport^15,16^ and is a figure of merit devised to allow comparisons of devices that have different magnetic field geometries. As the name implies, *ϵ*_eff_ will have the same value as the helical-ripple amplitude, *ϵ*_h_, for the limiting case in which this amplitude is a constant over the entire flux surface.

**Extended Data Fig. 1:**
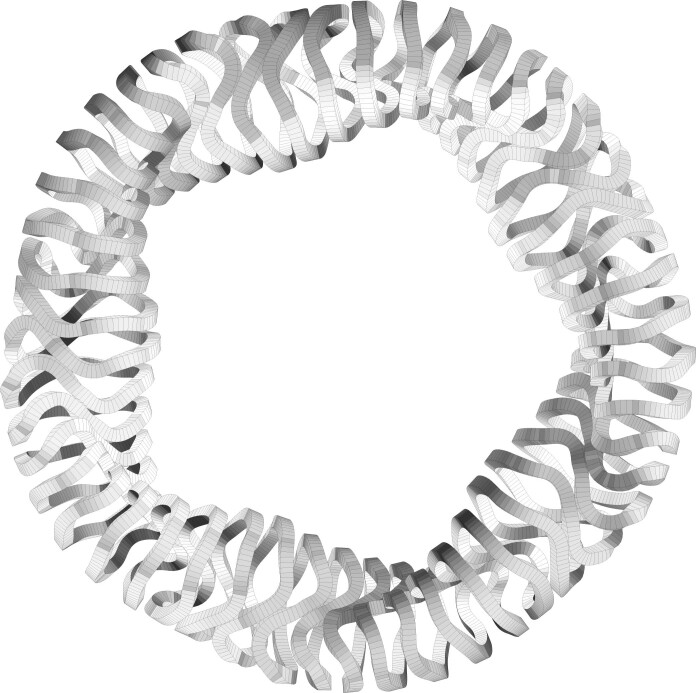
Non-planar coil system of W7-X, viewed from above. The complete set of superconducting coils also includes 20 planar coils, four in each field period, which are used to change the rotational transform and/or shift the plasma column; these are not shown as they remain current-free for the configurations considered here.

The temperature dependence of this result has long been considered a serious hindrance to the prospects of stellarator reactors, and clearly favours operation at the highest collisionality tolerable. High magnetic field strength and large aspect ratio, *R*_0_/*a* (*r* = *a* denotes the radius of the plasma’s last closed flux surface), are also beneficial but imply substantial capital cost for reactor construction as such expenditure scales with the stored magnetic energy of the device. The most ‘economical’ option offered by the 1/*ν* scalings is reduction of the effective helical ripple, which requires appropriate tailoring of the magnetic field so as to diminish the time-averaged radial drifts experienced by localized particles, and this has become a common goal of stellarator optimization since the inception of W7-X^17^. This device has five field periods (the integer number of times the magnetic field geometry repeats itself when going around the device once in the toroidal direction) and these periods are oriented such that the coil system appears nearly pentagonal when viewed from above (see Extended Data Fig. 1). The largest values of *B* are located in the pentagon’s ‘corners’, where the field curvature is particularly strong, but the majority of localized particles are thereby trapped in regions that have small **B** × ∇*B*, which reduces the associated radial drifts. Making use of this, W7-X was optimized for sufficiently reduced neoclassical energy transport that future HELIAS reactors with plasma volumes of 1,500 m^3^ become conceivable, requiring *ϵ*_eff_ values of a few per cent at most^18–20^.

In addition to causing 1/*ν* transport, localized particles are also responsible for the appearance of a radial electric field, *E*_*r*_, which must arise in stellarators to establish ambipolarity of the neoclassical particle fluxes (meaning that no net radial current flows in the plasma). This electric field introduces an **E** × **B** drift into the particles’ equation of motion, which causes localized particles to drift poloidally with a precession frequency *Ω*_*E*_ ≈ |*E*_*r*_|/(*rB*_0_), thereby placing an additional limit on the radial excursion of orbits. This poloidal precession is more important than collisions for particles that have *Ω*_*E*_ > *ν*_eff_, where *ν*_eff_ is the frequency with which collisional removal from the ripple occurs. For fusion plasmas, the collision frequency of electrons will exceed that of the ions by roughly two orders of magnitude and the ions will not be subject to 1/*ν* transport but will instead have their orbits constrained by *Ω*_*E*_, with a value of *E*_*r*_ such that the neoclassical ion particle flux is reduced to the value for electrons. For the interested reader, a more detailed presentation of neoclassical results is provided in Methods, where, in particular, it is demonstrated that strong reduction of electron 1/*ν* transport is also of direct benefit for reducing energy fluxes in the ion channel.

Given these ingredients, the recipe for reducing the sum of electron and ion neoclassical energy losses becomes readily apparent. Paramount is a minimization of electron 1/*ν* transport, which is best achieved in magnetic fields having small *ϵ*_eff_ and for plasmas at the highest tolerable collisionality. This strong reduction of the neoclassical electron energy transport is accompanied by a comparable decrease of the electron particle transport and ion neoclassical losses are then beneficially influenced by the ambipolar radial electric field. Reduction of ion transport is thus a knock-on effect relying on the actual realization of this predicted value of *E*_*r*_. Theoretically, such expectations are justified as particle transport is known to be intrinsically ambipolar for any turbulence satisfying the gyrokinetic orderings^21,22^, but experimental verification in W7-X is required for certainty.

**Fig. 1:**
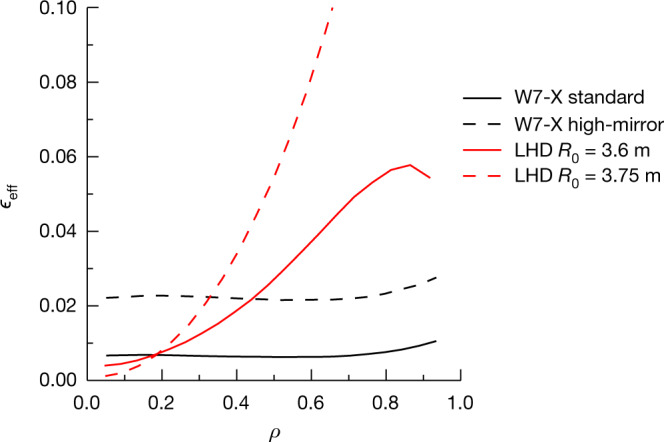
Radial profiles of the effective helical ripple. Radial profiles of *ϵ*_eff_ are shown for the W7-X standard (black continuous curve) and high-mirror (black broken curve) configurations as well as for the LHD *R*_0_ = 3.6 m (red continuous curve) and *R*_0_ = 3.75 m (red broken curve) configurations. In the last of these cases, the ‘missing’ portion of the curve that extends above the plot area increases roughly quadratically with normalized minor radius, *ρ* = *r*/*a*, to reach a value of 0.225 at *ρ* = 0.93.

In addition to reduced neoclassical transport, W7-X was also optimized for improved magnetohydrodynamic equilibrium and stability, negligible bootstrap current and good confinement of collisionless fast-particle orbits in plasmas with reactor-relevant pressure profiles^2^. The W7-X coil set was designed with a great deal of flexibility so as to provide access to a large configuration space, having candidates for which each of these goals is weighted to varying degrees^23^. Of particular interest for neoclassical transport studies is the portion of this space that has extremely small values of *ϵ*_eff_(*ρ* = *r*/*a*), an excellent example of which is the ‘standard’ configuration, which has equal currents in all five different types of non-planar coils. However, this configuration is expected to have larger bootstrap current and poorer fast-particle confinement than are desirable. Both deficiencies can be addressed by choosing a portion of the configuration space that has a larger variation of *B* along the magnetic axis. Such a ‘high-mirror’ configuration is achieved by adjusting the current ratios in non-planar coils of the same type so that these ratios are largest at the beginning of each field period (at the corners of the pentagon—see Extended Data Fig. 1) and smallest at its mid-point. The larger mirror term increases the fraction of trapped particles, however, leading to *ϵ*_eff_ values for the high-mirror configuration that exceed those of the standard configuration by factors of between 2.5 and 3.5, as can be seen by comparison of the radial profiles plotted in Fig. 1. These values are nevertheless small enough to pose no obstacle to reactor operation and, indeed, all HELIAS reactor studies are based on variants of the high-mirror configuration^19,20^.

Also shown in Fig. 1 are *ϵ*_eff_ profiles for two reference configurations of LHD^11^, a heliotron that has been in operation in Toki, Japan, since 1998. Such a comparison is a natural undertaking as W7-X and LHD are the largest members of the stellarator family currently in existence. These two devices have nearly the same plasma volume, although the aspect ratio of LHD (*R*_0_/*a* ≈ 6) is more compact than that of W7-X (*R*_0_/*a* ≳ 10). At LHD, vertical field coils can be used to shift the plasma column in the radial direction and configurations are typically differentiated according to the position of their major axis in vacuum; configurations with *R*_0_ = 3.6 m and *R*_0_ = 3.75 m are depicted here. As is typical for heliotrons, *ϵ*_eff_(*ρ*) is reduced by shifting the plasma inwards, whereas the case with the larger major radius is a good approximation to a stellarator field that has *ϵ*_eff_ ≈ *ϵ*_h_. Neoclassical transport coefficients for both LHD and W7-X have been calculated and benchmarked within an international collaboration^24^ and provide a basis for the calculations of neoclassical energy transport presented in the next section.

To visualize the benefit of optimizing the magnetic field geometry for reduced neoclassical transport, collisionless single-particle orbits of ions in W7-X standard and LHD *R*_0_ = 3.75 m are also provided here in two videos (see Supplementary Videos 1, 2).

## *Q*_neo_ for W7-X discharge 20180918.045

In many stellarator plasmas, the electron and ion temperatures are too low to cause neoclassical energy losses of a magnitude relevant for the plasma energy balance. As in tokamaks, this is attributed to turbulent transport, and the global energy confinement exhibited by the two concepts is quite comparable for devices of the same volume (see figure 36 of ref. ^25^). So far, plasma performance in W7-X is commonly limited by turbulence as well, and an experimental assessment of the neoclassical energy confinement in this device therefore requires a plasma scenario for which the turbulent transport is reduced.

**Extended Data Fig. 2:**
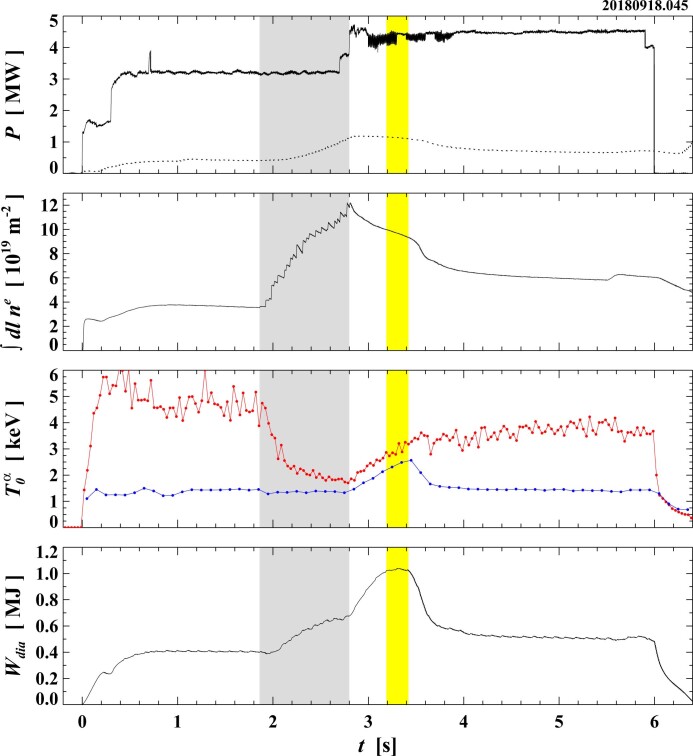
Time traces for W7-X discharge 20180918.045. In the top frame, the launched ECRH power is shown, as well as the radiated power measured by bolometers (dotted curve). The second frame plots the line-integrated density measured by an interferometer. The third frame depicts ‘core’ electron (red) and ion (blue) temperatures from Thomson scattering and XICS measurements, respectively. The diamagnetic energy trace during this discharge is given in the bottom frame. A series of 28 pellets is injected into the plasma at a frequency of 30 Hz during the time phase indicated in grey. *W*_dia_ values exceeding 1.02 MJ are recorded during the phase indicated in yellow.

In this regard, a substantial transient improvement of the energy confinement has been observed in certain W7-X experiments fuelled with hydrogen pellets^26,27^; the discharge 20180918.045, performed in the W7-X standard configuration, provides such an example. Time traces of interest for this experiment are provided in Extended Data Fig. 2, showing that a density ramp was initiated at *t* = 1.86 s by injecting a series of twenty-eight frozen hydrogen pellets into the plasma at a frequency of 30 Hz. In the aftermath of pellet fuelling the central density exceeds 10^20^ m^−3^ and the stored energy measured by a diamagnetic loop reaches a maximum value of *W*_dia_ = 1.02 MJ with central electron and ion temperatures both in excess of 2.5 keV, although the plasma is heated with only 4.5 MW of electron cyclotron resonance heating (ECRH). Taking fully ionized carbon as the predominant plasma impurity and a core value of the effective charge state equal to the global experimental value, *Z*_eff_ = 1.4, yields a central triple product of *n*^i^*T*^i^*τ*_E_ > 5.3 × 10^19^ m^−3^ keV s (where *n*^i^ and *T*^i^ indicate the density and temperature of ions, respectively), which is in the typical range of results achieved in W7-X during the high-performance phase of pellet-fuelled discharges^3,27^. The strong temperature dependence of stellarator neoclassical energy transport immediately raises the question of whether such record triple-product results are also experimental evidence for the reduction of neoclassical energy losses by optimization of the W7-X magnetic field. This discharge is particularly attractive for such an investigation as the 1.02 MJ are maintained for a full energy confinement time of 230 ms, thereby simplifying the plasma energy balance as the ∂*W*/∂*t* term appearing in this equation will be of negligible importance by the end of the high-energy phase. For interested readers, further details of this experiment can be found in Methods.

**Fig. 2:**
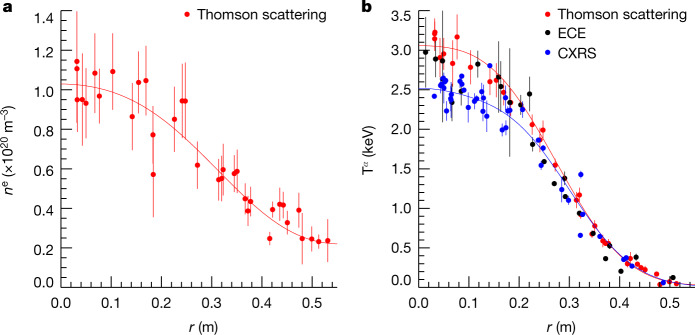
Density and temperature profiles for W7-X discharge 20180918.045 at *t* = 3.35 s. Thomson scattering measurements of *n*^e^ and *T*^e^ are shown by red data points, ECE results for *T*^e^ are plotted in black and CXRS values of *T*^i^ are given by blue circles. Error bars depict one standard deviation in the evaluation of the measurements. Fits to the experimental data used in the neoclassical analysis are depicted by the continuous curves with red used for the electron profiles and blue for the ions. The last closed flux surface of the equilibrium is at *r* = 0.508 m.

Calculation of the neoclassical losses requires knowledge of the plasma profiles. These are shown in Fig. 2 for *t* = 3.35 s at the end of the time period in which maximum *W*_dia_ is reached. The abscissa in these plots, *r*, is a flux-surface label, often referred to as the effective minor radius. The red data points depict *n*^e^ and *T*^e^, the density and temperature of electrons, respectively, obtained from Thomson scattering, and electron temperatures obtained with the electron cyclotron emission (ECE) system are shown in black. Values of *T*^*i*^ determined with charge exchange recombination spectroscopy (CXRS) are given by the blue circles. See Methods for information concerning these measurements and the error bars associated with them. Profile fits to these experimental data are depicted in Fig. 2 by continuous curves in red for electrons and in blue for ions. The stored energy associated with these profiles is *W* = 1.01 MJ, in good agreement with the experimentally measured *W*_dia_. To account for the uncertainty in experimental profiles, neoclassical results will also be determined here assuming certain variations of the plasma parameters, but, as will be seen, such sensitivity studies do not lead to fundamentally different conclusions.

Before proceeding to the analysis of these experimental results and their interpretation, it should be noted that two tenets underlie this process. First, that quantitative values of the neoclassical fluxes can be accurately determined for specified magnetic equilibria and plasma parameters, and second, that these fluxes represent the minimum value of transport that can be achieved for the given conditions. To substantiate the first of these claims, numerical means of solving the linearized drift kinetic equation and determining neoclassical transport coefficients have been thoroughly benchmarked as part of an international collaboration^24^. For the second, although there are various examples in the literature of stellarator confinement being consistent with neoclassical theory in the plasma core^25,28^, there are no published claims of experimental results for which the observed confinement exceeds neoclassical predictions.

**Fig. 3:**
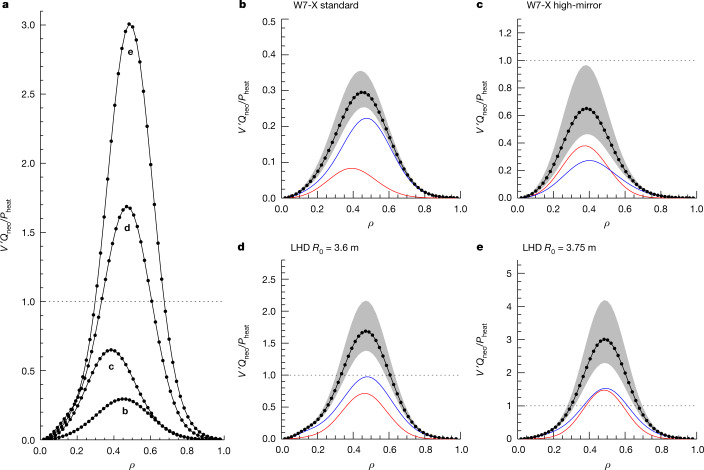
Comparison of the neoclassical energy fluxes associated with the high-temperature experimental conditions of discharge 20180918.045 at *t* = 3.35 s, for stellarator configurations with different degrees of neoclassical optimization. The plasma profiles used for this comparison are the fits of Fig. 2 and *P*_heat_ = 4.5 MW. **b**–**e**, Results are plotted for the configurations W7-X standard (**b**), W7-X high-mirror (**c**), LHD *R*_0_ = 3.6 m (**d**), and LHD *R*_0_ = 3.75 m (**e**). **a**, Radial profiles of the total neoclassical energy fluxes are provided for the four configurations (**b**–**e**) in a single plot to make direct comparison of the results straightforward. Physically impossible levels of the neoclassical energy fluxes are indicated by the appearance of regions with $V{}^{{\prime} }({Q}_{{\rm{n}}{\rm{e}}{\rm{o}}}^{{\rm{e}}}+{Q}_{{\rm{n}}{\rm{e}}{\rm{o}}}^{{\rm{i}}})/{P}_{{\rm{h}}{\rm{e}}{\rm{a}}{\rm{t}}} > 1$ (lying above the dotted horizontal line), which shows that the plasma profiles of Fig. 2 would not be attainable in the given configuration. More detailed results for each configuration are provided in the individual plots, where electron fluxes are shown in red, ion fluxes in blue and their sum appears as the black ball-and-chain curve. Also depicted are results for the sum of electron and ion fluxes obtained from a temperature-sensitivity study at constant pressure, that is, by replacing (*n*^e^, *T*^*α*^) with (*gn*^e^, *g*^−1^*T*^*α*^) and varying *g* from 0.9 (upper extent of the shaded region) to 1.1 (lower extent).

The importance of the neoclassical optimization for attaining record fusion-triple-product results in high-temperature stellarator plasmas can be demonstrated by calculating and comparing the neoclassical energy fluxes associated with the density and temperature profiles of Fig. 2 for configurations with different levels of optimization. This comparison is provided in Fig. 3a for the configurations (b) W7-X standard, (c) W7-X high-mirror, (d) LHD *R*_0_ = 3.6 m, and (e) LHD *R*_0_ = 3.75 m, and depicts the sum of electron and ion neoclassical energy fluxes, normalized to the heating power *P*_heat_ = 4.5 MW of discharge 20180918.045, as a function of normalized minor radius. Of primary interest here is to isolate the influence of the magnetic field configuration on the neoclassical results and, to this end, effects of the plasma volume on confinement have been removed by a slight linear upscaling of both LHD configurations so as to have the same plasma volume as W7-X; such scaling leaves the *ϵ*_eff_ profile and the aspect ratio unchanged. Influence of the magnetic field strength on the results is avoided by setting *B*_0_ = 2.5 T in all cases. This comparison demonstrates that the plasma profiles of discharge 20180918.045 in the W7-X standard configuration are only conceivable in magnetic configurations that have a comparably high level of neoclassical optimization. Indeed, the temperatures attained are high enough to imply a peak neoclassical energy flux nearly commensurate with the heating power for the W7-X high-mirror configuration, and for the LHD examples these energy fluxes exceed *P*_heat_ over a portion of the plasma and are thus physically impossible.

Further details of the neoclassical results are provided for each individual configuration in the remaining four frames of Fig. 3. In these plots, constituent contributions to the neoclassical energy fluxes made by electrons, ${Q}_{{\rm{neo}}}^{{\rm{e}}}$, and ions, ${Q}_{{\rm{neo}}}^{{\rm{i}}}$, are shown by the red and blue curves, respectively; their sum is again given by the black ball-and-chain curve. To illustrate the sensitivity of this sum to variations in plasma parameters, results for the total neoclassical energy fluxes are also depicted with (*n*^*e*^, *T*^*α*^) replaced by (*gn*^*e*^, *g*^−1^*T*^*α*^) and varying the scaling factor *g* in the range 0.9 ≤ *g* ≤ 1.1, where *α* = {e, i} indicates the values for electrons and ions, respectively). As the pressure profile is unaffected by this variation it is possible to perform all calculations using the same equilibrium. The values of collisionality remain sufficiently small in all these cases to ensure that the electron neoclassical fluxes are predominantly due to 1/*ν* transport. Given the strong temperature dependence of the losses in this regime, it is apparent that ${Q}_{{\rm{neo}}}^{{\rm{e}}}$ will attain its largest value for *g* = 0.9 and then decrease as *g* increases. The same behaviour is found for ${Q}_{{\rm{neo}}}^{{\rm{i}}}$ although the relative reduction is much weaker and can only be accounted for accurately by enforcing the ambipolarity constraint so as to obtain the correct value of the radial electric field. The sum ${Q}_{{\rm{neo}}}^{{\rm{e}}}+{Q}_{{\rm{neo}}}^{{\rm{i}}}$ is thus a monotonically decreasing function of *g* over the range of values considered and confirms the theoretical expectations mentioned in the previous section.

As expected qualitatively, the results of Fig. 3 confirm that $V{}^{{\prime} }({Q}_{{\rm{n}}{\rm{e}}{\rm{o}}}^{{\rm{e}}}+{Q}_{{\rm{n}}{\rm{e}}{\rm{o}}}^{{\rm{i}}})$ is an increasing function of *ϵ*_eff_ but quantification of this statement is far more difficult. For example, comparing the results of the two W7-X configurations, one finds ${Q}_{{\rm{neo}}}^{{\rm{e}}}$ larger in the high-mirror case by a factor of five, which corresponds well with the ${{\epsilon }}_{{\rm{eff}}}^{3/2}$ dependence of 1/*ν* transport. However, the total neoclassical energy flux for W7-X high-mirror is increased by a factor only somewhat larger than two, owing to the much smaller increase in ${Q}_{{\rm{neo}}}^{{\rm{i}}}$. This example also demonstrates the nonlinearity of the neoclassical fluxes—quantitative accuracy in the determination of these fluxes cannot rely only on comparisons of *ϵ*_eff_.

With regard to the higher levels of neoclassical energy transport for the LHD configurations, it should be emphasized that these are provided only to give the reader some idea of how large neoclassical energy fluxes can become at high *T*^*α*^ in stellarator-type devices that were not explicitly optimized to reduce neoclassical transport. Such observations have been accounted for in contemporary designs of heliotron reactors by shifting the plasma column further inwards and also adjusting the pitch modulation of the helical windings so as to obtain a tolerable level of neoclassical transport for the envisaged plasma conditions^29^.

**Extended Data Fig. 3:**
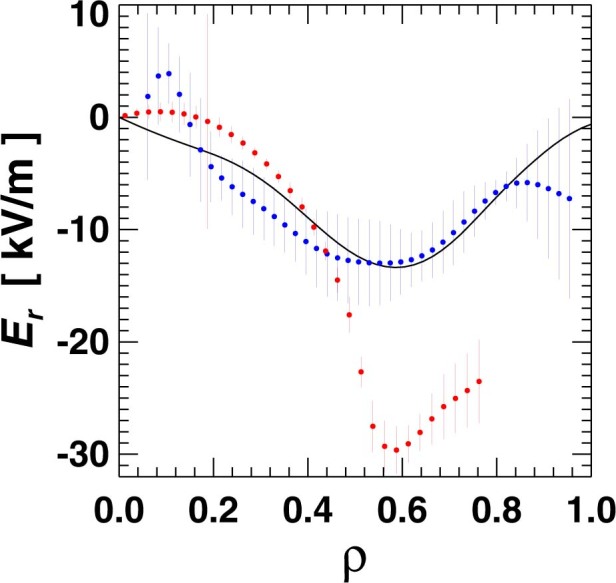
Profiles of the radial electric field as a function of the normalized plasma radius. Experimental results from CXRS (blue) and XICS (red) measurements are compared to the theoretical expectations from the ambipolarity constraint (black) for the plasma profiles of Fig. 2. Error bars depict one standard deviation in the evaluation of the measurements. The XICS analysis is described in ref. ^30^; documentation of the methodology used to evaluate the CXRS measurements is in preparation.

As explained in the previous section, the structure of *B* is critical in determining the level of electron neoclassical transport and the reduction of losses in the ion channel relies on the appearance of the radial electric field needed to satisfy the ambipolarity constraint. Experimental profiles of *E*_*r*_ obtained from X-ray imaging crystal spectrometer (XICS) measurements have been published for other examples of pellet-fuelled discharges in W7-X and have been shown to conform with neoclassical predictions^30^. The same claim can be made for discharge 20180918.045, which has *E*_*r*_ profiles obtained from CXRS in addition to those from XICS (see Extended Data Fig. 3). With this additional information, plasma profiles leading to large values of the triple product in W7-X high-temperature plasmas may be taken as experimental evidence for the reduction of neoclassical losses through an appropriate optimization of the confining magnetic field. In particular, the very small values of *ϵ*_eff_(*ρ*), which characterize the W7-X standard configuration, are indispensable for achievement of the high electron and ion temperatures of discharge 20180918.045 with a heating power of only 4.5 MW. As the behaviour of the bootstrap current in W7-X has already been shown to conform with neoclassical expectations^31^, reduction of all neoclassical fluxes and flows in line with the W7-X optimization goals is also substantiated.

Confirming the importance of the W7-X neoclassical optimization for improving plasma performance is only a first step, however, as high-triple-product phases have been of limited duration in the device so far. It is postulated that—owing to properties of the turbulent transport^32^—such performance requires the establishment and sustainment of sufficiently steep density gradients. A similar tendency was also observed in the preceding Wendelstein experiment, W7-AS, where the appearance of ‘optimum confinement’ conditions was always accompanied by such gradients^25^. These W7-AS discharges were heated using a combination of ECRH and neutral beam injection (NBI), the latter providing the plasma with a strong central particle source, while simultaneously the edge particle source due to recycling neutrals dropped to unusually low levels. Under such conditions, steep density gradients could be maintained throughout the 250-ms heating pulse but, although long in comparison to *τ*_E_, such pulses were insufficient to claim ‘steady-state’ conditions as the sinks due to neutral-particle pumping by plasma-facing components did not saturate during this time. At W7-X, NBI was successfully commissioned during the second portion of the 2017–2018 campaign and will allow future investigations into whether optimum confinement conditions can also be realized in this device during the 10-s duration of NBI pulses. To truly test the steady-state perspectives of the HELIAS concept, the ECRH at W7-X has been designed to provide the plasma with 1,800 s of continuous-wave power and a water-cooled, high-heat-flux divertor is currently being installed in the device to provide the necessary particle and heat exhaust over this period of time. If steep density gradients are indeed the key to improving the confinement of W7-X plasmas, density profile tailoring over such time scales will probably need to rely on the capabilities of a new steady-state pellet injector, which should also go into operation during the next campaign.

## Methods

### Theoretical details

In high-temperature stellarator plasmas, the radial components of the flux-surface-averaged neoclassical particle and energy flux densities (*Γ*_neo_ and *Q*_neo_, respectively) of particle species *α* may be expressed as^24^

1 $$[\begin{array}{c}{\varGamma }_{{\rm{n}}{\rm{e}}{\rm{o}}}^{\alpha }\\ {Q}_{{\rm{n}}{\rm{e}}{\rm{o}}}^{\alpha }/{T}^{\alpha }\end{array}]=-{n}^{\alpha }{L}_{11}^{\alpha }\left\{[\begin{array}{c}1\\ {\delta }_{21}^{\alpha }\end{array}]\,\left(\frac{1}{{n}^{\alpha }}\frac{{\rm{d}}{n}^{\alpha }}{{\rm{d}}r}-\frac{{q}^{\alpha }{E}_{r}}{{T}^{\alpha }}\right)+[\begin{array}{c}{\delta }_{12}^{\alpha }\\ {\delta }_{22}^{\alpha }\end{array}]\frac{1}{{T}^{\alpha }}\frac{{\rm{d}}{T}^{\alpha }}{{\rm{d}}r}\right\},$$

where *n* = *n*(*r*), *T* = *T*(*r*) and *q* are the density, temperature and charge of the given species, respectively, *E*_*r*_ = *E*_*r*_(*r*) is the radial electric field and the *δ*_*ij*_ are normalized transport coefficients

$${\delta }_{12}=\frac{{L}_{12}}{{L}_{11}}-\frac{3}{2},\,{\delta }_{21}=\frac{{L}_{21}}{{L}_{11}},\,{\delta }_{22}=\frac{{L}_{22}}{{L}_{11}}-\frac{3{L}_{21}}{2{L}_{11}},$$

comprising appropriate combinations of elements of the neoclassical transport matrix

$${L}_{ij}=\frac{2}{\sqrt{{\rm{\pi }}}}\underset{0}{\overset{\infty }{\int }}{\rm{d}}K\sqrt{K}{h}_{i}{h}_{j}\,{{\rm{e}}}^{-K}D(K),{\rm{with}}\,{h}_{1}=1,{h}_{2}=K$$

where *K* ≡ *κ*/*T* = *mv*^2^/(2*T*) is the normalized kinetic energy and *D* is the so-called mono-energetic radial transport coefficient. The terminology ‘radial’ is used here to denote quantities that are oriented perpendicularly to flux surfaces so that the radial coordinate *r* should be understood as a flux-surface label.

To understand how the neoclassical energy transport scales with various plasma and configuration parameters, it is sufficient to consider a simple heuristic description of random-walk diffusion processes having *D* ∝ ℱ(Δ*r*)^2^*ν*_eff_ where ℱ is the fraction of particles participating in the process, Δ*r* is the characteristic step size of such particles and *ν*_eff_ is the frequency with which a step is taken. For the cases of interest here, the transport is due to the pitch-angle scattering portion of the linearized collision operator, allowing one to express the ‘effective’ step frequency as *ν*_eff_ = *ν*/ℱ^2^ where *ν* is the 90°-deflection frequency and the ℱ^−2^ enhancement accounts for the fact that scattering through the portion of phase space comprising ℱ occurs more often than scattering through 90°. The heuristic expression for the transport coefficient then simplifies to *D* ∝ (Δ*r*)^2^*ν*/ℱ leaving only Δ*r* and ℱ to be determined.

The variation of **B** along field lines in toroidal devices causes a **B** × ∇*B* drift of guiding centre particle trajectories in this field, having a characteristic velocity *v*_d_ = *κ*/(|*q*|*R*_0_*B*_0_) (refs. ^6,7^). For particles localized in a stellarator’s helical ripple, this drift will lead to a displacement from the flux surface by an amount Δ*r* = min(*v*_d_/*ν*_eff_, *v*_d_/*Ω*_*E*_), depending on whether the effective collision frequency, *ν*_eff_, or the **E** × **B** precessional frequency, *Ω*_*E*_ = |*E*_*r*_|/(*rB*_0_), is larger for the given particle. The first of these cases is typical for electrons in a high-temperature stellarator plasma and will be considered in the remainder of this paragraph. For the simplest of stellarator magnetic fields having a constant helical-ripple amplitude over the entire flux surface, *ϵ*_h_ = *ϵ*_h_(*r*), the fraction of localized particles scales as ${\mathcal F} \propto {{\epsilon }}_{{\rm{h}}}^{1/2}$ and the heuristic expression for the radial transport coefficient then yields $D\propto {{\epsilon }}_{{\rm{h}}}^{3/2}{v}_{{\rm{d}}}^{2}/\nu$. Substituting this result into the formula for the *L*_*ij*_ and taking the collision frequency proportional to *nv*^−3^, one recovers the scaling ${L}_{ij}\propto {{\epsilon }}_{{\rm{h}}}^{3/2}{T}^{7/2}{n}^{-1}{({R}_{0}{B}_{0})}^{-2}$. This result can be generalized to arbitrary stellarator fields if one replaces *ϵ*_h_ with the appropriate value of *ϵ*_eff_, which explains why this latter quantity is given the name ‘effective helical ripple for 1/*ν* transport’. To derive the scalings of the neoclassical energy flux in the 1/*ν* regime, one approximates *V*′ ∝ *rR*_0_ and the inverse gradient scale lengths appearing in the braces of equation (1) as *a*^−1^ to obtain $V{}^{{\prime} }{Q}_{{\rm{n}}{\rm{e}}{\rm{o}}}\propto {{\epsilon }}_{{\rm{e}}{\rm{f}}{\rm{f}}}^{3/2}{T}^{9/2}{R}_{0}^{-1}{B}_{0}^{-2}$.

For a fusion plasma having equal species temperatures the collision frequency of electrons will exceed that of the ions by roughly two orders of magnitude. Consequently, for localized ions the characteristic radial step size of the diffusive random-walk process will be Δ*r* ∝ *v*_d_/*Ω*_*E*_ and ℱ will combine the fractions of phase space in which particle orbits change their trapping states due to either collisions or drifts. For the stellarator field with *ϵ*_h_ = *ϵ*_h_(*r*), the former case is characterized by a ‘collisional boundary layer’ of width^13^ ${{\epsilon }}_{{\rm{h}}}^{1/2}{({\nu }_{{\rm{eff}}}/{{\Omega }}_{E})}^{1/2}={(\nu /{{\Omega }}_{E})}^{1/2}$ whereas the latter is signified by ℱ_tr_, the fraction of ‘transition’ orbits^33^, which is a largely geometric factor reflecting the topology of the local maxima of *B*. Substitution of these quantities into the heuristic expression for the transport coefficient then yields for the ions $D\propto {({v}_{{\rm{d}}}/{\varOmega }_{E})}^{2}\nu {(\sqrt{\nu /{\varOmega }_{E}}+{ {\mathcal F} }_{{\rm{t}}{\rm{r}}})}^{-1}$ which is more commonly found in the literature as two separate results for the limiting cases in which one of the terms within the parentheses is far larger than its counterpart, the so-called √*ν* regime with $D\propto {v}_{{\rm{d}}}^{2}\,{(\nu /{\varOmega }_{E}^{3})}^{1/2}$ and the ‘*ν* regime’ with $D\propto {({v}_{{\rm{d}}}/{{\Omega }}_{E})}^{2}\nu /{ {\mathcal F} }_{{\rm{tr}}}$. Using ${\mathscr{A}}$ to signify those parameters in *ν*/*Ω*_*E*_ that are not of direct relevance to plasma-parameter scalings, the neoclassical ion energy losses are then seen to obey

$$V{}^{{\prime} }{Q}_{{\rm{n}}{\rm{e}}{\rm{o}}}\propto \frac{{n}^{2}\,{T}^{3/2}}{{R}_{0}\,{E}_{r}^{2}}{\left\{{\left(\frac{{\mathscr{A}}n{B}_{0}}{|{E}_{r}|{T}^{3/2}}\right)}^{1/2}+{ {\mathcal F} }_{{\rm{t}}{\rm{r}}}\right\}}^{-1}.$$

Although this expression is conveniently compact, it leaves the complicated dependence of *E*_*r*_ on plasma and device parameters unspecified. This will be addressed next for the case of most relevance to the reduction of neoclassical transport in W7-X.

Unlike the axisymmetric tokamak, neoclassical particle fluxes are not intrinsically ambipolar in a stellarator, and thus a theoretical means of determining the *E*_*r*_ profile is provided by enforcing the ambipolarity constraint. In a pure hydrogen plasma (using *α* = e to denote electrons and *α* = i for ions) for which *n*^e^ = *n*^i^ = *n* and for which *q*^e^ = −*e* and *q*^i^ = *e*, where *e* is the elementary charge, equating ${{\Gamma }}_{{\rm{neo}}}^{{\rm{e}}}$ and ${{\Gamma }}_{{\rm{neo}}}^{{\rm{i}}}$ will yield

$$\frac{e{E}_{r}}{{T}^{\alpha }}=\frac{{T}^{{\rm{e}}}{T}^{{\rm{i}}}}{{T}^{\alpha }({L}_{11}^{{\rm{e}}}{T}^{{\rm{i}}}+{L}_{11}^{{\rm{i}}}{T}^{{\rm{e}}})}\left\{({L}_{11}^{{\rm{i}}}-{L}_{11}^{{\rm{e}}})\frac{1}{n}\frac{{\rm{d}}n}{{\rm{d}}r}+{L}_{11}^{{\rm{i}}}{\delta }_{12}^{{\rm{i}}}\frac{1}{{T}^{{\rm{i}}}}\frac{{\rm{d}}{T}^{{\rm{i}}}}{{\rm{d}}r}-{L}_{11}^{{\rm{e}}}{\delta }_{12}^{{\rm{e}}}\frac{1}{{T}^{{\rm{e}}}}\frac{{\rm{d}}{T}^{{\rm{e}}}}{{\rm{d}}r}\right\}.$$

Substituting this result back into the neoclassical expressions, one obtains for the particle flux density

$${{\Gamma }}_{{\rm{neo}}}^{\alpha }=-\frac{n({T}^{{\rm{e}}}+{T}^{{\rm{i}}}){L}_{11}^{{\rm{e}}}{L}_{11}^{{\rm{i}}}}{({L}_{11}^{{\rm{e}}}{T}^{{\rm{i}}}+{L}_{11}^{{\rm{i}}}{T}^{{\rm{e}}})}\left\{\frac{1}{n}\frac{{\rm{d}}n}{{\rm{d}}r}+\frac{{\delta }_{12}^{{\rm{e}}}}{({T}^{{\rm{e}}}+{T}^{{\rm{i}}})}\frac{{\rm{d}}{T}^{{\rm{e}}}}{{\rm{d}}r}+\frac{{\delta }_{12}^{{\rm{i}}}}{({T}^{{\rm{e}}}+{T}^{{\rm{i}}})}\frac{{\rm{d}}{T}^{{\rm{i}}}}{{\rm{d}}r}\right\},$$

and for the energy flux densities

$$\begin{array}{c}{Q}_{{\rm{neo}}}^{\alpha }=-\frac{n{L}_{11}^{{\rm{e}}}{T}^{{\rm{e}}}{L}_{11}^{{\rm{i}}}{T}^{{\rm{i}}}}{({L}_{11}^{{\rm{e}}}{T}^{{\rm{i}}}+{L}_{11}^{{\rm{i}}}{T}^{{\rm{e}}})}\{{\delta }_{21}^{\alpha }\frac{({T}^{{\rm{e}}}+{T}^{{\rm{i}}})}{{T}^{\beta }}\frac{1}{n}\frac{{\rm{d}}n}{{\rm{d}}r}+{\delta }_{12}^{\beta }{\delta }_{21}^{\alpha }\frac{1}{{T}^{\beta }}\frac{{\rm{d}}{T}^{\beta }}{{\rm{d}}r}\\ +\left[({\delta }_{22}^{\alpha }-{\delta }_{12}^{\alpha }{\delta }_{21}^{\alpha })\frac{{L}_{11}^{\alpha }}{{L}_{11}^{\beta }}+{\delta }_{22}^{\alpha }\frac{{T}^{\alpha }}{{T}^{\beta }}\right]\frac{1}{{T}^{\alpha }}\frac{{\rm{d}}{T}^{\alpha }}{{\rm{d}}r}\},\end{array}$$

where the species indices are chosen to be [*α*, *β*] = [e, i] or [i, e] as appropriate. However, one should recall that the ${L}_{ij}^{{\rm{i}}}$ are dependent on *E*_*r*_, so that profitable use of these equations requires that special circumstances hold. One such example is the fusion-relevant case that has *T*^e^ = *T*^i^ = *T* for which the radial electric field equation becomes

$$\frac{e{E}_{r}}{T}={\left(1+\frac{{L}_{11}^{{\rm{e}}}}{{L}_{11}^{{\rm{i}}}}\right)}^{-1}\left\{\left(1-\frac{{L}_{11}^{{\rm{e}}}}{{L}_{11}^{{\rm{i}}}}\right)\frac{1}{n}\frac{{\rm{d}}n}{{\rm{d}}r}+\left({\delta }_{12}^{{\rm{i}}}-\frac{{L}_{11}^{{\rm{e}}}}{{L}_{11}^{{\rm{i}}}}{\delta }_{12}^{{\rm{e}}}\right)\frac{1}{T}\frac{{\rm{d}}T}{{\rm{d}}r}\right\},$$

and will yield *E*_*r*_ ∝ *T* for the limiting case in which electron 1/*ν* transport has been sufficiently reduced to satisfy ${L}_{11}^{{\rm{e}}}/{L}_{11}^{{\rm{i}}}\ll 1$. In the same limit, the particle flux density is well approximated by

$${{\Gamma }}_{{\rm{neo}}}^{\alpha }\approx -n{L}_{11}^{{\rm{e}}}\left\{\frac{2}{n}\frac{{\rm{d}}n}{{\rm{d}}r}+({\delta }_{12}^{{\rm{e}}}+{\delta }_{12}^{{\rm{i}}})\frac{1}{T}\frac{{\rm{d}}T}{{\rm{d}}r}\right\},$$

making it evident that electrons are the rate-controlling species. Additionally, one notices that the factor outside the braces in ${Q}_{{\rm{neo}}}^{\alpha }$ simplifies to $nT{L}_{11}^{{\rm{e}}}$ and that the largest normalized transport coefficients associated with the temperature gradients are ${\delta }_{22}^{{\rm{e}}}+{\delta }_{12}^{{\rm{i}}}{\delta }_{21}^{{\rm{e}}}$ for electrons and $({\delta }_{22}^{{\rm{i}}}-{\delta }_{12}^{{\rm{i}}}{\delta }_{21}^{{\rm{i}}}){L}_{11}^{{\rm{i}}}/{L}_{11}^{{\rm{e}}}$ for ions. At first glance, the largeness of ${L}_{11}^{{\rm{i}}}/{L}_{11}^{{\rm{e}}}$ might lead one to expect a considerable difference in the size of these two terms, but the strong temperature dependence of 1/*ν* transport makes the ${\delta }_{ij}^{{\rm{e}}}$ considerably larger than their ${\delta }_{ij}^{{\rm{i}}}$ counterparts. Indeed, under the assumption of ‘pure’ regimes, the *L*_*ij*_ may be expressed in terms of the so-called gamma function, and one finds ${\delta }_{12}^{{\rm{e}}}=7/2$, ${\delta }_{21}^{{\rm{e}}}=5$ and ${\delta }_{22}^{{\rm{e}}}=45/2$  whereas  $1/2\le {\delta }_{12}^{{\rm{i}}}\le 5/4$, $2\le {\delta }_{21}^{{\rm{i}}}\le 11/4$  and  $3\le {\delta }_{22}^{{\rm{i}}}\le 99/16$ where the smaller values hold in the *ν* regime and the larger for the √*ν* regime^34^. When these limits apply one obtains $25\le {\delta }_{22}^{{\rm{e}}}+{\delta }_{12}^{{\rm{i}}}{\delta }_{21}^{{\rm{e}}}\le 115/4$ and $2\le {\delta }_{22}^{{\rm{i}}}-{\delta }_{12}^{{\rm{i}}}{\delta }_{21}^{{\rm{i}}}\le 11/4$ so that the *δ*_*ij*_ combination of relevance for electrons is an order of magnitude larger than its counterpart for ions. The neoclassical energy transport of electrons and ions will thus be of similar magnitudes and the strong reduction of ${L}_{11}^{{\rm{e}}}$ is clearly seen to be of benefit to both species.

### Details concerning the calculation of neoclassical energy fluxes

For the magnetic configurations considered in this work all equilibria have been determined with the Variational Moments Equilibrium Code (VMEC)^35^ and subsequently expressing the results in terms of Boozer flux coordinates^36^. This provides all information concerning the magnetic field needed as input by the Drift Kinetic Equation Solver (DKES)^37^, which is used to prepare a dataset of mono-energetic transport coefficients covering the entire range of ν* and *E*_*r*_ values relevant for determining the *L*_*ij*_ given any combination of density and temperature producing the pressure profile of the VMEC equilibrium. The radial electric field profile is determined self-consistently by using numerical root-finding techniques to determine solutions to the nonlinear ambipolarity constraint.

For the W7-X calculations, equilibria for the pressure profile of discharge 20180918.045 at *t* = 3.35 s have been used, whereas the LHD results presented here have been obtained for vacuum equilibria. The LHD equilibria thus ignore the outward shift of the plasma column due to non-zero plasma pressure—the so-called Shafranov shift^38^—which leads to an increase of the neoclassical energy losses^39,40^. This deleterious effect can be counteracted to an extent by using the vertical field coils to shift the plasma axis back to its vacuum position, but a deformation in the shape of flux surfaces remains, which serves to degrade the neoclassical confinement. The neoclassical energy losses calculated here for LHD are thus ‘best case’ results. By contrast, the W7-X optimization had the explicit goal of using finite-plasma-pressure effects to its benefit; for the high-mirror configuration, *ϵ*_eff_ decreases monotonically as the pressure increases, whereas for the standard configuration, small-to-modest pressure has little influence on this quantity.

The results plotted in Fig. 3 also allow a rough assessment of the reactor prospects for these four configurations with regard to their neoclassical energy confinement. For a deuterium-tritium plasma meant to produce a power of 3 gigawatts thermal (GWth) and that has 600 MW of *α*-particle heating, it is necessary to increase the temperatures of discharge 20180918.045 by a factor of six and the density by a factor of three, assuming that reactor-sized versions of W7-X and LHD have their dimensions increased by a factor of four and their magnetic field strength doubled. Scaling up the configurations leaves *ϵ*_eff_ unchanged so that the electron 1/*ν* energy fluxes will increase according to ${T}^{9/2}{R}_{0}^{-1}{B}_{0}^{-2}$, and thus by a factor of roughly 200. Scaling the maximum electron energy fluxes of Fig. 3 by this factor, one obtains 75 MW for W7-X standard, 340 MW for W7-X high-mirror, 640 MW for LHD *R*_0_ = 3.6 m and 1,320 MW for LHD *R*_0_ = 3.75 m. For stellarator reactor plasmas, ion neoclassical energy fluxes are at least as large as those of the electrons but even the somewhat extreme assumption of ${Q}_{{\rm{neo}}}^{{\rm{i}}}=2{Q}_{{\rm{neo}}}^{{\rm{e}}}$ would make the total neoclassical energy fluxes only a fraction of the *α*-particle heating for W7-X standard. At first glance, the reactor prospects of W7-X high-mirror appear questionable, but one must realize that this back-of-the-envelope estimate ignores changes to the configuration due to increased plasma pressure. In the current example, the normalized plasma pressure, *β* ∝ *nTB*^−2^, is increased by a factor of 4.5 and, when this is accounted for, *ϵ*_eff_ in the high-mirror configuration is reduced by enough that ${Q}_{{\rm{neo}}}^{{\rm{e}}}$ would drop by a factor of two. For the LHD cases it is necessary to choose different scaling factors; the *R*_0_ = 3.6 m case becomes viable with a factor five larger size and the somewhat reduced temperature that this allows. (This assumes that the vertical field coils of the heliotron are used to compensate the Shafranov shift as the neoclassical transport in such a high-pressure equilibrium would otherwise become intolerable).

It should also be pointed out that the optimization of W7-X was undertaken roughly thirty years ago and that great improvements in the projected fast-particle confinement of HELIAS have been made in the intervening years. Such improvements have simultaneously reduced the *ϵ*_eff_ values from the per cent level of W7-X to the per mille level in new reactor candidates, thereby decreasing neoclassical energy transport for prospective fusion plasmas to very small levels. This development, coupled with the predominance of turbulent transport in the W7-X experiment, have focused recent theoretical and numerical efforts on the further optimization of the HELIAS concept to also contend with this transport channel. Such efforts are still in their infancy, however, and it is not yet possible to foresee what combination of optimized magnetic field and plasma conditions will best reduce turbulent transport and what influence such a combination will have on the other HELIAS optimization goals.

### Background and experimental details

Since its inception, W7-X has been conceived as a means of demonstrating the attractive properties that advanced stellarators offer as prospective fusion reactors. Ultimately, this envisions not only the achievement of high-triple-product plasma operation but the ability to maintain such operation over time scales far in excess of those characterizing all physical processes of the plasma and the plasma-facing components. To enable such ‘steady-state’ scenarios, electron cyclotron resonance heating (ECRH) of W7-X plasmas is available using ten gyrotrons, each of which is capable of providing continuous-wave power over a time interval of 1,800 s (ref. ^41^). The gyrotron frequency of 140 GHz is resonant at the second harmonic for a magnetic field strength of *B* = 2.5 T with cutoff densities of *n*_c_ = 1.2 × 10^20^ m^−3^ for waves with extraordinary-mode (X2) polarization and 2*n*_c_ for ordinary-mode (O2) polarization. At these densities and for W7-X plasma volumes of nearly 30 m^3^ the collisional transfer of energy from electrons to ions should be excellent, so that *T*^i^ ≈ *T*^e^ can be expected in spite of lacking a direct means of heating the ions. Such operational conditions also mimic qualitatively those of a reactor where the heating power of fusion *α*-particles goes chiefly to electrons, which subsequently heat deuterium and tritium ions by collisional energy exchange.

With regard to plasma-facing components, steady-state operation of W7-X will only become possible after the installation of a water-cooled, high-heat-flux (HHF) divertor capable of providing the necessary particle and power exhaust throughout the course of such experiments. The installation of this divertor with all its intricate plumbing requirements was started at the end of 2018, with completion scheduled for the end of 2021. The experimental results considered here were obtained in the latter portion of 2018, during the second half of a campaign in which initial experience with divertor operation in W7-X was acquired through the use of an uncooled test divertor unit (TDU) having the same geometry that its HHF successor will have. Pellet fuelling was already observed to improve plasma performance during the first portion of this TDU campaign^3,26^ and such results motivated further investigations once wall conditioning had been greatly improved following ‘boronization’ of the device, early in the second half of the TDU campaign. On many occasions, considerable increases of the plasma diamagnetic energy, *W*_dia_, were measured in the aftermath of pellet fuelling and the discharge 20180918.045 is used here to illustrate such results.

Time traces for this discharge are plotted in Extended Data Fig. 2. Following a short start-up phase in X-mode to achieve plasma breakdown, the ECRH power (first plot in the figure) is launched exclusively with O-mode polarization so as to avoid large amounts of reflected power should pellet fuelling lead transiently to densities above the X2 cutoff. Also shown by the dotted curve is the radiated power measured with bolometer arrays; this power originates in roughly equal portions from the confined plasma and from the region outside the last closed flux surface, which includes the large magnetic islands that are the basis of the W7-X divertor concept. Initially, the line-integrated electron density, ∫d*ℓn*^e^, is held at a value of roughly 3 × 10^19^ m^2^, which is too low to promote effective collisional energy transfer from electrons to ions (where the d*ℓ* appearing in the integrand is the differential length along the line of sight). Consequently, the 3.2 MW of ECRH used during this phase produces plasmas that have *T*^e^ ≫ *T*^i^ centrally as the core temperature measurements from Thomson scattering and the X-ray imaging crystal spectrometer (XICS) diagnostics show (third plot in the figure). A density ramp is initiated 1.86 s into the discharge by injecting a series of 28 frozen hydrogen pellets into the plasma at a frequency of 30 Hz using a so-called blower gun^42^. Although the fuelling efficiency of the first few pellets is poor, noticeable improvement occurs thereafter^26^ and increments in the line-integrated density caused by individual pellets become clearly discernible with ∫d*ℓn*^e^ slightly exceeding 12 × 10^19^ m^−2^ when the series ends at *t* = 2.8 s. At this time point, the ECRH power is increased to 4.5 MW to maintain *T*^e^ at values sufficient for good O2 absorption. This is further aided at W7-X by a multi-pass launch scheme which uses specially prepared reflecting surfaces to redirect unabsorbed ECRH power back into the plasma. With this scheme, ray-tracing simulations predict that more than 95% of the launched ECRH power will be deposited in the plasma during the high-performance phase of discharge 20180918.045, a value that has been confirmed by the analysis of stray-radiation measurements from the experiment taken with ‘sniffer’ probes.

The pellets obviously provide the plasma with a particle source but their thermalization also introduces a considerable energy sink into the electron energy balance particularly as the thermalization process also strongly heats the ions for the given initial conditions^43^. In response, *T*^e^ falls and the central electron temperature has dropped below 2 keV—nearly to the level of central *T*^i^—when the increase in ECRH power occurs. To this time point, pellet fuelling has been accompanied by a rise in the diamagnetic energy from *W*_dia_ = 0.40 MJ at *t* = 1.8 s to *W*_dia_ = 0.68 MJ at *t* = 1.8 s. With the larger heating power, the time rate of change of *W*_dia_ then increases considerably and the diamagnetic energy attains its maximum value of 1.02 MJ after another 400 ms. In the absence of pellet fuelling, the line-integrated density decreases throughout this time, making it evident that a temperature increase must also have taken place and, indeed, the central values exceed 2.5 keV for both electrons and ions. Thus, an experimental situation has arisen that has high density and high temperatures simultaneously, a situation ideal for testing the efficacy of the W7-X optimization regarding reduction of the neoclassical transport. The discharge shown here is particularly attractive for such an investigation as the 1.02 MJ are maintained for 230 ms which corresponds to one energy confinement time given the 4.5 MW of ECRH used to heat the plasma. This simplifies considerations of the energy balance as the ∂*W*/∂*t* term appearing in this equation will be of negligible importance by the end of the high-energy phase.

ECRH power deposition was not highly localized for discharge 20180918.045, as the electron cyclotron waves were launched with ordinary-mode polarization. Nevertheless, the great majority of the heating power is deposited within *ρ* < 0.4 at the time point of the analysis. At this radius, and in the neighbouring vicinity at least out to *ρ* = 0.55, the sum of electron and ion neoclassical energy fluxes exceeds 25% of the heating power and is thus of relevance when considering the energy balance. A larger share of this balance remains unaccounted for, however, even after deducting the observed energy losses due to radiation. This shortfall indicates that turbulent transport must still be of importance for explaining the experimental results, especially in the plasma periphery where low temperatures lead to negligible neoclassical energy transport. In spite of the level of turbulent transport needed to fully explain this discharge, the high temperatures achieved are nevertheless responsible for an experimentally relevant neoclassical energy flux, even though the W7-X standard configuration has extremely small values of the effective helical ripple. Assuming such temperatures had been achieved in configurations without a comparably high level of neoclassical optimization quickly leads to the physically impossible result of neoclassical energy fluxes exceeding the heating power, as is demonstrated in the comparison of Fig. 3.

### Diagnostics

The plasma energy has been measured with a diamagnetic loop, located in the (toroidal) symmetry plane of one of the five field periods^44^. It encircles the plasma and is equipped with four compensation coils, which are also located inside the vacuum vessel and directly attached to the diamagnetic loop itself. These do not encircle the plasma and can therefore be used to compensate measurements of the main loop for errors due to eddy currents in the adjacent vacuum vessel as well as fluctuations of externally driven currents in the main superconducting magnetic field coils.

The line-integrated electron density was determined by a single-channel dispersion interferometer employing a CO_2_ laser measuring at a fundamental wavelength of 10.6 μm (ref. ^45^). The probing beam passes through the plasma twice by making use of a corner cube reflector; the single-pass path length through the plasma is roughly 1.3 m. The statistical error for ∫d*ℓn*^e^ is generally given as 10^18^ m^−2^ and there was an additional systematic error ≤4 × 10^18^ m^−2^ during the portion of the experimental campaign during which discharge 20180918.045 was performed.

The Thomson scattering system at W7-X employs three YAG lasers to provide full profiles of electron density and temperature at a rate of 30 Hz. Scattered light is collected by two in-vessel optical systems and routed to polychromators outside the torus hall via optical fibres. Forty-two spatial channels are available, each employing five interference filters to provide spectral resolution^46,47^. Spectral calibration is done ex vessel by withdrawing the optical systems and illuminating a diffuse-reflecting screen with a supercontinuum light source^46^. Absolute calibration for density measurements was obtained by performing anti-Stokes rotational Raman scattering in nitrogen. Bayesian analysis is used to determine the most probable electron temperature and density for each Thomson scattering volume, as well as their uncertainties and cross-correlation. Error bars in Fig. 2 depict the width of the 95% confidence interval divided by four, which corresponds to the one-standard-deviation interval of a normal distribution; see ref. ^47^ for full details.

Electron cyclotron emission is used at W7-X to determine the electron temperature using a 32-channel heterodyne radiometer probing X2 emission^48^. The *T*^e^ data plotted in Fig. 2 give ECE values of the electron temperature averaged over a centred 20-ms time window, with the error bars depicting the standard deviation of these values and also accounting for systematic calibration uncertainties, largely due to the unknown thermal drifts of the radiometer sensitivity. An optimized line-of-sight is realized across the 3D plasma shape by means of an in vessel Gauss telescope. The radiometer channels are calibrated relative to each other and absolute calibration is carried out using a second ‘identical’ Gauss telescope outside the torus that views a hot-cold light source^49^. For the results presented here, the absolute calibration has been scaled up by a factor of 1.58 to account for the relative mismatch with Thomson data on the day of the experiment. Direct interpretation of the radiometer signals as a local temperature measurement would require that ideal blackbody conditions be fulfilled in the plasma, whereas in reality, non-thermal ‘hot’ components of the electron distribution function appear in the spectrum as well. A further complication arises in semi-transparent plasmas owing to a degradation of spatial resolution. The *T*^e^ profile is therefore inferred by applying Bayesian analysis with forward modelling of the radiation transport in the plasma; for full details, see ref. ^50^.

The CXRS diagnostic at W7-X makes use of a high-étendue spectrometer to provide measurements of *T*^i^ at 51 spatial locations as well as *E*_*r*_ at 25 of these. Given the prevalence of carbon in the device’s plasma-facing components, this element is always present as an impurity in W7-X plasmas and the visible charge exchange line C^VI^ at 529.07 nm provides the strongest possible signal. Active charge exchange measurements are enabled by using short ‘blips’ of neutral beam injection (NBI) for diagnostic purposes and subtracting the passive spectrum observed before and after the NBI from this data. A complete description may be found in ref. ^51^.

The XICS diagnostic is based on spectroscopic analysis of emission from highly charged argon impurities that are seeded into the plasma in trace amounts for diagnostic purposes^52^. The XICS system records a 1D image of line-integrated spectra, from which the ion temperature is found by measuring the Doppler-broadened width of the emission lines. The local plasma parameters are found by using a tomographic inversion based on a known VMEC equilibrium^53^. Detailed descriptions of the XICS diagnostic on W7-X can be found in ref. ^54^ although improvements in the diagnostic analysis have since been implemented, including compensation for spherical aberrations and the sub-pixel distribution of photons on the detector. At the time point analysed for this discharge, the XICS values of *T*^i^ exceed those of CXRS by 150 to 200 eV and indicate *T*^i^ > *T*^e^ for *r* > 0.2 m despite the fact that no direct heating of the ions occurs using ECRH. This is at odds with energy balance considerations, however, which argue for *T*^i^ ≈ *T*^e^ outside the region of power deposition. These expectations are better fulfilled by the CXRS data and, given the strong dependence of the neoclassical losses on temperature, it has therefore been decided to err on the side of caution by using only the CXRS values of *T*^i^ for the profile fits used in the calculations of neoclassical fluxes. The time evolution of the *T*^i^ profile measured by CXRS has also been confirmed through the measurements of the XICS system.

Plasma radiation is measured by two bolometer cameras—each with arrays of detectors that have a 5 μm blackened gold-foil absorber—installed in one of the mid-field-period toroidal-symmetry planes of W7-X, and with spatial resolution of between 3 and 4 cm (ref. ^55^). Line-integrated signals from 65 channels are used to obtain radiation intensity distributions by tomographic reconstruction with ‘relative gradient smoothing’ as regularization functional (to be published). Flux-surface-averaged radial emissivity profiles are then derived by averaging these 2D-emissivity distributions in the poloidal direction. The total radiated power loss is a linear interpolation of radiation from the observation volume to that of the entire plasma volume. Toroidal variations of the radiation strength are not considered, an assumption supported by the results of edge modelling.

## Online content

Any methods, additional references, Nature Research reporting summaries, source data, extended data, supplementary information, acknowledgements, peer review information; details of author contributions and competing interests; and statements of data and code availability are available at 10.1038/s41586-021-03687-w.

## Supplementary information

Supplementary Information This file contains detailed notes regarding Supplementary Videos 1 and 2.

Video 1 Localized ion trajectory in the W7-X standard configuration – see Supplementary Information document for detailed description.

Video 2 Localized ion trajectory in the LHD configuration having a major radius of 3.75 m - see Supplementary Information document for detailed description.

## Data Availability

The data depicted in the plots of this paper and other findings of this study are available from the corresponding author upon reasonable request.
