# Publisher Correction: Demonstration of reduced neoclassical energy transport in Wendelstein 7-X

**DOI:** 10.1038/s41586-021-04023-y

**Published:** 2021-10-12

**Authors:** C. D. Beidler, H. M. Smith, A. Alonso, T. Andreeva, J. Baldzuhn, M. N. A. Beurskens, M. Borchardt, S. A. Bozhenkov, K. J. Brunner, H. Damm, M. Drevlak, O. P. Ford, G. Fuchert, J. Geiger, P. Helander, U. Hergenhahn, M. Hirsch, U. Höfel, Ye. O. Kazakov, R. Kleiber, M. Krychowiak, S. Kwak, A. Langenberg, H. P. Laqua, U. Neuner, N. A. Pablant, E. Pasch, A. Pavone, T. S. Pedersen, K. Rahbarnia, J. Schilling, E. R. Scott, T. Stange, J. Svensson, H. Thomsen, Y. Turkin, F. Warmer, R. C. Wolf, D. Zhang, the W7-X Team, I. Abramovic, S. Äkäslompolo, J. Alcusón, P. Aleynikov, K. Aleynikova, A. Ali, A. Alonso, G. Anda, E. Ascasibar, J. P. Bähner, S. G. Baek, M. Balden, M. Banduch, T. Barbui, W. Behr, A. Benndorf, C. Biedermann, W. Biel, B. Blackwell, E. Blanco, M. Blatzheim, S. Ballinger, T. Bluhm, D. Böckenhoff, B. Böswirth, L.-G. Böttger, V. Borsuk, J. Boscary, H.-S. Bosch, R. Brakel, H. Brand, C. Brandt, T. Bräuer, H. Braune, S. Brezinsek, K.-J. Brunner, R. Burhenn, R. Bussiahn, B. Buttenschön, V. Bykov, J. Cai, I. Calvo, B. Cannas, A. Cappa, A. Carls, L. Carraro, B. Carvalho, F. Castejon, A. Charl, N. Chaudhary, D. Chauvin, F. Chernyshev, M. Cianciosa, R. Citarella, G. Claps, J. Coenen, M. Cole, M. J. Cole, F. Cordella, G. Cseh, A. Czarnecka, K. Czerski, M. Czerwinski, G. Czymek, A. da Molin, A. da Silva, A. de la Pena, S. Degenkolbe, C. P. Dhard, M. Dibon, A. Dinklage, T. Dittmar, P. Drewelow, P. Drews, F. Durodie, E. Edlund, F. Effenberg, G. Ehrke, S. Elgeti, M. Endler, D. Ennis, H. Esteban, T. Estrada, J. Fellinger, Y. Feng, E. Flom, H. Fernandes, W. H. Fietz, W. Figacz, J. Fontdecaba, T. Fornal, H. Frerichs, A. Freund, T. Funaba, A. Galkowski, G. Gantenbein, Y. Gao, J. García Regaña, D. Gates, B. Geiger, V. Giannella, A. Gogoleva, B. Goncalves, A. Goriaev, D. Gradic, M. Grahl, J. Green, H. Greuner, A. Grosman, H. Grote, M. Gruca, O. Grulke, C. Guerard, P. Hacker, X. Han, J. H. Harris, D. Hartmann, D. Hathiramani, B. Hein, B. Heinemann, P. Helander, S. Henneberg, M. Henkel, U. Hergenhahn, J. Hernandez Sanchez, C. Hidalgo, K. P. Hollfeld, A. Hölting, D. Höschen, M. Houry, J. Howard, X. Huang, Z. Huang, M. Hubeny, M. Huber, H. Hunger, K. Ida, T. Ilkei, S. Illy, B. Israeli, S. Jablonski, M. Jakubowski, J. Jelonnek, H. Jenzsch, T. Jesche, M. Jia, P. Junghanns, J. Kacmarczyk, J.-P. Kallmeyer, U. Kamionka, H. Kasahara, W. Kasparek, N. Kenmochi, C. Killer, A. Kirschner, T. Klinger, J. Knauer, M. Knaup, A. Knieps, T. Kobarg, G. Kocsis, F. Köchl, Y. Kolesnichenko, A. Könies, R. König, P. Kornejew, J.-P. Koschinsky, F. Köster, M. Krämer, R. Krampitz, A. Krämer-Flecken, N. Krawczyk, T. Kremeyer, J. Krom, I. Ksiazek, M. Kubkowska, G. Kühner, T. Kurki-Suonio, P. A. Kurz, M. Landreman, P. Lang, R. Lang, S. Langish, H. Laqua, R. Laube, S. Lazerson, C. Lechte, M. Lennartz, W. Leonhardt, C. Li, C. Li, Y. Li, Y. Liang, C. Linsmeier, S. Liu, J.-F. Lobsien, D. Loesser, J. Loizu Cisquella, J. Lore, A. Lorenz, M. Losert, A. Lücke, A. Lumsdaine, V. Lutsenko, H. Maaßberg, O. Marchuk, J. H. Matthew, S. Marsen, M. Marushchenko, S. Masuzaki, D. Maurer, M. Mayer, K. McCarthy, P. McNeely, A. Meier, D. Mellein, B. Mendelevitch, P. Mertens, D. Mikkelsen, A. Mishchenko, B. Missal, J. Mittelstaedt, T. Mizuuchi, A. Mollen, V. Moncada, T. Mönnich, T. Morisaki, D. Moseev, S. Murakami, G. Náfrádi, M. Nagel, D. Naujoks, H. Neilson, R. Neu, O. Neubauer, T. Ngo, D. Nicolai, S. K. Nielsen, H. Niemann, T. Nishizawa, R. Nocentini, C. Nührenberg, J. Nührenberg, S. Obermayer, G. Offermanns, K. Ogawa, J. Ölmanns, J. Ongena, J. W. Oosterbeek, G. Orozco, M. Otte, L. Pacios Rodriguez, N. Panadero, N. Panadero Alvarez, D. Papenfuß, S. Paqay, E. Pawelec, T. S. Pedersen, G. Pelka, V. Perseo, B. Peterson, D. Pilopp, S. Pingel, F. Pisano, B. Plaum, G. Plunk, P. Pölöskei, M. Porkolab, J. Proll, M.-E. Puiatti, A. Puig Sitjes, F. Purps, M. Rack, S. Récsei, A. Reiman, F. Reimold, D. Reiter, F. Remppel, S. Renard, R. Riedl, J. Riemann, K. Risse, V. Rohde, H. Röhlinger, M. Romé, D. Rondeshagen, P. Rong, B. Roth, L. Rudischhauser, K. Rummel, T. Rummel, A. Runov, N. Rust, L. Ryc, S. Ryosuke, R. Sakamoto, M. Salewski, A. Samartsev, E. Sánchez, F. Sano, S. Satake, J. Schacht, G. Satheeswaran, F. Schauer, T. Scherer, A. Schlaich, G. Schlisio, F. Schluck, K.-H. Schlüter, J. Schmitt, H. Schmitz, O. Schmitz, S. Schmuck, M. Schneider, W. Schneider, P. Scholz, R. Schrittwieser, M. Schröder, T. Schröder, R. Schroeder, H. Schumacher, B. Schweer, S. Sereda, B. Shanahan, M. Sibilia, P. Sinha, S. Sipliä, C. Slaby, M. Sleczka, W. Spiess, D. A. Spong, A. Spring, R. Stadler, M. Stejner, L. Stephey, U. Stridde, C. Suzuki, V. Szabó, T. Szabolics, T. Szepesi, Z. Szökefalvi-Nagy, N. Tamura, A. Tancetti, J. Terry, J. Thomas, M. Thumm, J. M. Travere, P. Traverso, J. Tretter, H. Trimino Mora, H. Tsuchiya, T. Tsujimura, S. Tulipán, B. Unterberg, I. Vakulchyk, S. Valet, L. Vanó, P. van Eeten, B. van Milligen, A. J. van Vuuren, L. Vela, J.-L. Velasco, M. Vergote, M. Vervier, N. Vianello, H. Viebke, R. Vilbrandt, A. von Stechow, A. Vorköper, S. Wadle, F. Wagner, E. Wang, N. Wang, Z. Wang, T. Wauters, L. Wegener, J. Weggen, T. Wegner, Y. Wei, G. Weir, J. Wendorf, U. Wenzel, A. Werner, A. White, B. Wiegel, F. Wilde, T. Windisch, M. Winkler, A. Winter, V. Winters, S. Wolf, R. C. Wolf, A. Wright, G. Wurden, P. Xanthopoulos, H. Yamada, I. Yamada, R. Yasuhara, M. Yokoyama, M. Zanini, M. Zarnstorff, A. Zeitler, H. Zhang, J. Zhu, M. Zilker, A. Zocco, S. Zoletnik, M. Zuin

**Affiliations:** 1 Max-Planck-Institut für Plasmaphysik, Greifswald, Germany; 2 Laboratorio Nacional de Fusion, CIEMAT, Madrid, Spain; 3 Laboratory for Plasma Physics (LPP), École royale militaire/Koninklijke Militaire School (ERM/KMS), Brussels, Belgium; 4 Princeton Plasma Physics Laboratory, Princeton, NJ USA; 5 Present Address: Fritz-Haber-Institut der Max-Planck-Gesellschaft, Berlin, Germany; 6 Wigner Research Centre for Physics, Budapest, Hungary; 7 Massachusetts Institute of Technology, Cambridge, MA USA; 8 Max Planck Institute for Plasma Physics, Garching, Germany; 9 University of Wisconsin Madison, Madison, WI USA; 10 Institute for Energy and Climate Research – Plasma Physics, Research Center Jülich, Jülich, Germany; 11 The Australian National University, Canberra, Australian Capital Territory Australia; 12 Technical University of Denmark, Kongens Lyngby, Denmark; 13 Eindhoven University of Technology, Eindhoven, Netherlands; 14 University of Cagliary, Cagliari, Italy; 15 Consorzio RFX, Padova, Italy; 16 Instituto de Plasmas e Fusao Nuclear, Lisbon, Portugal; 17 CEA Cadarache, Saint-Paul-lez-Durance, France; 18 Ioffe Physical-Technical Institute of the Russian Academy of Sciences, St Petersburg, Russian Federation; 19 Oak Ridge National Laboratory, Oak Ridge, TN USA; 20 University of Salerno, Fisciano, Italy; 21 ENEA Centro Ricerche Frascati, Frascati, Italy; 22 Institute of Plasma Physics and Laser Microfusion, Warsaw, Poland; 23 University of Szczecin, Szczecin, Poland; 24 University of Milano-Bicocca, Milan, Italy; 25 Auburn University, Auburn, AL USA; 26 Karlsruhe Institute of Technology, Eggenstein-Leopoldshafen, Germany; 27 National Institute for Fusion Science, Toki, Japan; 28 Universidad Carlos III de Madrid, Madrid, Spain; 29 Greifswald University, Greifswald, Germany; 30 Institute for Surface Process Engineering and Plasma Technology, University of Stuttgart, Stuttgart, Germany; 31 Austrian Academy of Science, Vienna, Austria; 32 Institute for Nuclear Research, Kiev, Ukraine; 33 Technical University of Berlin, Berlin, Germany; 34 University of Opole, Opole, Poland; 35 Aalto University, Espoo, Finland; 36 University of Maryland, College Park, MA USA; 37 Physikalisch Technische Bundesanstalt (PTB), Braunschweig, Germany; 38 Kyoto University, Kyoto, Japan; 39 Istituto di Fisica del Plasma “Piero Caldirola”, Milan, Italy; 40 Culham Centre for Fusion Energy, Abingdon, UK; 41 Los Alamos National Laboratory, Los Alamos, NM USA

Correction to: *Nature* 10.1038/s41586-021-03687-wPublished online 11 August 2011

In the original version of this Article, in the fifth paragraph of the section titled “*Q*neo for W7-X discharge 20180918.045,” errors appeared in reference to the configurations shown in the curves of Fig. 3a and associated Fig. 3b−e panels. In the sentence originally reading “This comparison is provided in **Fig. 3a** for the configurations (**Fig. 3b**) W7-X standard, (**Fig. 3c**) W7-X high-mirror, (**Fig. 3d**) LHD R0 = 3.6 m, and (**Fig. 3e**) LHD R0 = 3.75 m....,” the references to Fig. 3b−e should instead have referred to the lettered curves “b,c,d,e” shown in the graph of Fig. 3a. The text has now been amended to read: “This comparison is provided in **Fig. 3a** for the configurations (b) W7-X standard, (c) W7-X high-mirror, (d) LHD R0 = 3.6 m, and (e) LHD R0 = 3.75 m….”

Additionally, a member of the W7-X Team was incorrectly listed as “M. Sanchez”; their name has been corrected to “E. Sánchez.”

The original Article has been corrected online.

