# Supplementary material for: Demonstration of reduced neoclassical energy transport in Wendelstein 7-X
Source: Nature. 2021 Aug 11;596(7871):221–6. doi: 10.1038/s41586-021-03687-w (PMC8357633; doi:10.1038/s41586-021-03687-w)
Supplement: Supplementary file 1 — This file contains detailed notes regarding Supplementary Videos 1 and 2. [file 41586_2021_3687_MOESM1_ESM.pdf]

nature portfolio

https://doi.org/10.1038/s41586-021-03687-w

Supplementary information

Demonstration of reduced neoclassical energy transport in Wendelstein 7-X

In the format provided by the authors and unedited

Guiding-centre orbits of localised ions in W7-X and LHD — Two videos depict collisionless guiding-centre orbits of ions in the W7-X standard and LHD $R_0 = 3.75$ m configurations in the presence of a radial electric field assumed to have the magnitude $E_r = -4T/(ea)$, where T is the ion temperature (expressed in eV), e is the elementary charge and a denotes the value of the flux-surface label, r, on the last-closed-flux-surface of the magnetic equilibrium. At their launch point, the particles are further assumed to have parallel and perpendicular velocity components of $v_\parallel = 0.1v_{th}$ and $v_\perp = 1.5v_{th}$, respectively, where $v_{th} = (2T/m)^{1/2}$ is the thermal velocity and m is the ion mass; the kinetic energy of the particles is thus $\kappa = mv^2/2 = 2.26\,T$. The guiding-centre trajectory of the ion is traced in red and shown from various viewpoints: the entire torus is visible at the upper left, while in the lower row two field periods that the orbit traverses are blown up and viewed from above (left) and below (right). The variation of B on a "reference" flux surface having $\rho^2 \equiv (r/a)^2 = 0.5$ is shown using a color scale — particle reflection occurs at the points along the trajectory for which B becomes large enough that $v_\parallel = 0$. On the upper right, the projection of the orbit onto a plane of constant toroidal angle is shown using a coordinate system in which flux surfaces are circular; the reference flux surface is depicted here in yellow and $r = a$ is shown in gray. When drifts of localised particles are constrained by a radial electric field, the characteristic scale length for the departure of particles' drift surfaces from their flux surfaces is given by $\Delta r = v_d/\Omega_E$, where $v_d = \kappa/(eR_0B_0)$ is the magnitude of the radial drift velocity and and $\Omega_E = |E_r|/(rB_0)$ is the $\mathbf{E} \times \mathbf{B}$ precessional frequency. In these expressions, R_0 is the major radius of the torus and B_0 is the strength of the magnetic field. Expressed in dimensionless form, the scale length for the orbits depicted here becomes $\Delta r/a = 0.565\,\rho/A$, where $A = R_0/a$ is the plasma aspect ratio. The closer coincidence of drift and flux surfaces seen in the video for W7-X is thus attributable to a combination of two factors — first, the optimisation of the magnetic field geometry placing localised particles in regions with small $\mathbf{B} \times \nabla B$ and, second, the larger aspect ratio of W7-X ($A \approx 11$) compared to LHD ($A \approx 6.25$). As both factors are also of relevance for $1/\nu$ transport (the former for determining ϵ_{eff}, and the latter appearing explicitly in the scaling of the energy flux), the particle orbits shown are also indicative for the improvement of neoclassical confinement in this regime.
